# Treatment of Bing–Neel syndrome with first line sequential chemoimmunotherapy: A case report

**DOI:** 10.1097/MD.0000000000017794

**Published:** 2019-11-01

**Authors:** Maria Gavriatopoulou, Ioannis Ntanasis-Stathopoulos, Lia-Angela Moulopoulos, Alexandros Manaios, Despina Fotiou, Evangelos Eleutherakis-Papaiakovou, Magdalini Migkou, Charis Bourgioti, Evangelos Terpos, Efstathios Kastritis, Meletios-Athanasios Dimopoulos

**Affiliations:** a Department of Clinical Therapeutics, National and Kapodistrian University of Athens, School of Medicine, Alexandra General Hospital; b First Department of Radiology, National and Kapodistrian University of Athens, School of Medicine, Areteion Hospital; c “Athens Vision” Ophthalmology Clinic, Athens, Greece.

**Keywords:** Bing–Neel, ibrutinib, methotrexate, rituximab, Waldenström macroglobulinemia

## Abstract

**Rationale:**

Bing–Neel syndrome (BNS) is a rare manifestation of Waldenström macroglobulinemia (WM) with <200 cases reported in the literature. Herein, we describe a case of newly diagnosed BNS treated with a novel therapeutic strategy.

**Patient concerns:**

A 67-year-old woman diagnosed with asymptomatic WM 3 years ago presented with gradual vision deterioration the past 3 months. Ophthalmologic examination revealed bilateral reduction in visual acuity (7/10) and bilateral optic disc swelling which was more prominent in the left eye.

**Diagnoses:**

Brain imaging revealed bilateral swelling of optic nerves extending from the retina to the optic chiasm and swelling of the left optic tract. Patchy enhancement of optic nerves was also shown upon intravenous contrast administration. Flow cytometry of the cerebrospinal fluid (CSF) revealed the presence of κ-light chain restricted, monoclonal B-lymphocytes. CSF protein electrophoresis showed a monoclonal band in the gamma region and immunofixation was positive for immunoglobulin M and kappa light chain. Thus, the diagnosis of BNS was established.

**Interventions:**

The patient was initially treated with intrathecal methotrexate and systemic chemotherapy. Following 2 intrathecal methotrexate infusions, CSF flow cytometry did not detect any cells, whereas the patient reported improvement in visual acuity. Therefore, we opted to start maintenance treatment with IV rituximab and per os ibrutinib.

**Outcomes:**

Following 1 year posttreatment initiation, visual problems have resolved completely and the patient remains on hematologic and imaging complete response.

**Lessons:**

We propose a novel sequential chemoimmunotherapy approach for BNS treatment aiming both at rapid disease control and deep and durable remission with minimization of induced toxicity.

## 1 Introduction

Bing–Neel syndrome (BNS) is a rare manifestation of Waldenström macroglobulinemia (WM) with <200 cases reported in the literature. Although the recent years there has been a significant effort in formulating clinical practice guidelines, still diagnosis and management remains challenging.^[1]^ BNS is characterized by the malignant invasion of lymphoplasmacytic cells in the central nervous system (CNS), including brain parenchyma, spinal cord, meninges, and cerebrospinal fluid (CSF). It may present as a disease relapse among previously treated patients with WM or as the initial manifestation of symptomatic disease even in the absence of other criteria for symptomatic WM, as in our case.^[1,2]^ From its initial description by Bing and von Neel in 1936,^[3]^ the literature reports of the disease are scarce, whereas the 4 largest multicenter case-series encompass 24 to 44 patients.^[2,4–6]^ Although the incidence of BNS is difficult to be determined precisely, a retrospective cohort including more than 1500 patients with WM suggested a prevalence of <1%.^[7]^ Clinical presentation varies significantly among BNS cases ranging from locomotor and sensory deficits to neuropsychiatric disorders, and it depends on the affected CNS compartment.^[1,6]^ Hyperviscosity syndrome, immunoglobulin M (IgM)-related neuropathies, WM transformation to high-grade lymphoma or de novo lymphomas evading CNS may have overlapping clinical signs and symptoms with BNS and, thus, they should be ruled out during the diagnostic workup.^[1]^ Magnetic resonance imaging (MRI) is recommended and may be highly suggestive of CNS invasion; interestingly, optic nerve involvement has been rarely reported.^[5]^ Although biopsy is considered the gold standard for definitive diagnosis, CSF analysis supporting the presence of lymphoplasmacytic lymphoma is also acceptable.^[1]^ Herein, we describe a case of newly diagnosed BNS treated with a novel therapeutic strategy. In our case, CSF flow cytometry and immunofixation showing monoclonal B-cells and IgM paraprotein with the same kappa-light chain restriction as in the serum and the bone marrow established BNS diagnosis. In compliance with the CARE guidelines, patient informed consent has been obtained and all reported clinical and imaging data have been de-identified.

## 2 Case description

**Figure 1:**
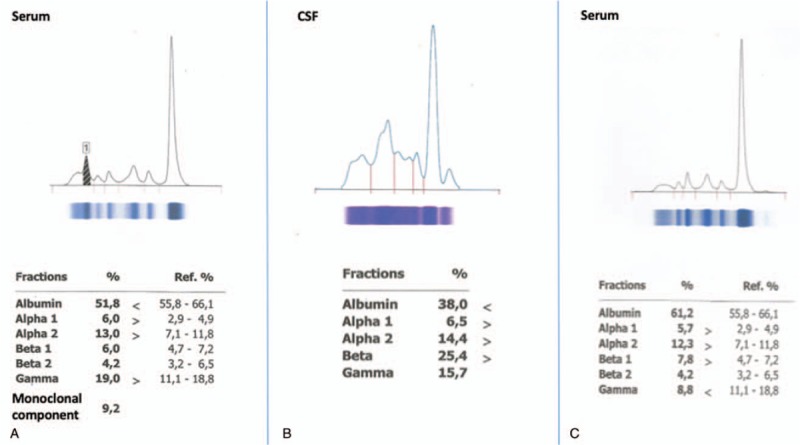
(A) Serum electrophoresis before treatment initiation demonstrating a monoclonal M-paraprotein of 0.61 g/dL. (B) Cerebrospinal fluid (CSF) electrophoresis showing a monoclonal component in the gamma region; immunofixation revealed immunoglobulin M and kappa light-chain monoclonal bands. (C) Serum electrophoresis after 6 months of treatment initiation showing disappearance of the monoclonal band, whereas serum immunofixation was negative.

**Figure 2:**
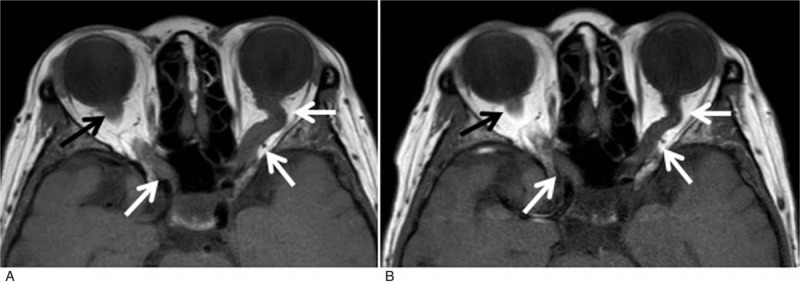
(A) At presentation brain magnetic resonance imaging revealed bilateral swelling of optic nerves extending from the retina to the optic chiasm and swelling of the left optic tract. T1-weighted image in the axial plane shows diffuse enlargement of both optic nerves (arrows). (B) Corresponding T1-weighted image in the axial plane of the same patient 3 months after treatment demonstrates decrease in the size of both nerves.

A 67-year-old woman diagnosed with asymptomatic WM 3 years ago presented with gradual vision deterioration the past 3 months. Ophthalmologic examination revealed bilateral reduction in visual acuity (7/10) and bilateral optic disc swelling which was more prominent in the left eye. Clinical examination was otherwise unremarkable. The patient reported no weight loss or B-symptoms. At the time of initial WM diagnosis, the bone marrow biopsy showed 15% infiltration of M(κ)-clonal lymphoplasmacytic cells and the serum immunofixation was positive for monocloncal IgM, while serum IgM levels were 147 mg/dL. Laboratory examinations were within normal ranges with no signs of anemia or thrombocytopenia, whereas clinical and imaging assessment did not reveal any sites of lymphadenopathy or hepatosplenomegaly. Therefore, the patient was defined as asymptomatic and had been under follow-up every 4 months. Two years postinitial diagnosis a new diagnostic bone marrow biopsy revealed an increase in clonal infiltration up to 40% and genetic tests performed both in the bone marrow and in the serum cell-free DNA did not detect any MYD88 or CXCR4 mutations. The presence of mutations in the aforementioned genes was evaluated by allele-specific polymerase chain reaction and direct sequencing, as previously described.^[8]^ Serum IgM levels had been gradually increasing as well with no other concurrent symptomatology and reached at 723 mg/dL at the time of presentation with visual loss (serum M-peak = 0.61 g/dL) (Fig. 1A). Her medical history was also remarkable of right eye cataract surgery, thyroidectomy, and T4 hormone replacement therapy, but she had no history of migraine. She was also current smoker with mild hypercholesterolemia. Due to the presence of risk factors for cardiovascular disease, we performed initially an extensive workup including electrocardiogram, cardiac ultrasound, sonography of the carotid, and vertebral arteries that revealed no clinically significant abnormalities. Coagulation studies were within normal limits. No serum autoantibodies were detected. Apart from an erythrocyte sedimentation rate of 140 mm at 1st hour, there were no other clinical signs of giant cell arteritis. Although the IgM was relatively low, we evaluated serum viscosity, which was 2.0 cp and, thus, we ruled out hyperviscosity syndrome. MRI of brain revealed bilateral swelling of optic nerves extending from the retina to the optic chiasm and swelling of the left optic tract. Patchy enhancement of optic nerves was also shown upon intravenous contrast administration (Fig. 2A). MRI of the spine indicated no abnormalities. Subsequently, we performed a lumbar puncture; flow cytometry of the CSF revealed the presence of κ-light chain restricted, monoclonal B-lymphocytes accounting for the 86% of CD45 positive cells along with CD3(+)CD4(+) and CD3(+)CD8(+) T-lymphocytes. CSF protein electrophoresis showed a monoclonal band in the gamma region and immunofixation was positive for IgM and kappa light chain (Fig. 1B). MYD88 status was indeterminate. Thus, the diagnosis of BNS was established. Patient was initially treated with intrathecal methotrexate 15 mg twice weekly and systemic chemotherapy with HyperCVAD. However, after the course 1 of the 1st cycle she experienced grade 3 neutropenic fever that eventually resolved with granulocyte colony-stimulating factor and intravenous antibiotics. Following 2 intrathecal methotrexate infusions, CSF flow cytometry did not detect any cells, whereas the patient reported improvement in visual acuity. Therefore, we opted to start treatment with IV rituximab 375 mg/m^2^ every 4 weeks and per os ibrutinib 420 mg daily. Six months posttreatment initiation the patient has great visual improvement with resolution of papilledema and bilateral optic nerve swelling (Fig. 2B), whereas IgM levels have been reduced to 25.6 mg/dL, the serum protein electrophoresis (Fig. 1C) and immunofixation are negative for monoclonal component and bone marrow biopsy shows no evidence of disease infiltration. Therefore, the patient has achieved clinical, hematologic, and radiologic complete response. No adverse events have been reported except for transient diarrhea grade 1 probably related to ibrutinib. After completing 1 year treatment with rituximab, the patient discontinued rituximab and remains on ibrutinib monotherapy in complete response and with no major adverse events.

## 3 Discussion

The principal aim of BNS therapeutics is patients’ clinical improvement and progression-free survival (PFS) prolongation irrespectively of the possible persistence of the lymphoplasmacytic clone or MRI findings.^[1]^ Due to the rarity of the disease, no universal treatment guidelines have been formulated yet; thus, the therapeutic strategy is personalized and depends on patient characteristics and comorbidities, prior lines of therapy, the involved CNS compartment and the availability of treatment regimens. It may include systemic and/or intrathecal chemotherapy, radiotherapy, and targeted therapies.^[1]^ The reported response rates among patients receiving traditional chemotherapy regimens with or without rituximab reach at 70%, whereas a response rate of 85% has been recently reported with ibrutinib monotherapy.^[2,4,6]^ Interestingly, exposure to rituximab was associated with improved overall survival in 1 retrospective study.^[4]^ Ibrutinib has been shown to penetrate the blood–brain barrier^[9]^ and has shown significant clinical efficacy as monotherapy among 28 BNS cases in a multicenter case series study^[6]^ and 9 case reports.^[9–15]^ In our case, we initially chose to administrate high-dose intrathecal methotrexate along with systemic chemotherapy that resulted in rapid improvement in symptoms and disappearance of malignant clonal cells in the CSF. Subsequently, based on the reported efficacy results of both rituximab and ibrutinib we decided to switch therapy to a less toxic chemofree regimen. Taking into consideration the benefit mentioned earlier from rituximab and ibrutinib administration for patients with BNS, along with the improved PFS of patients with WM receiving ibrutinib-rituximab in the phase 3 iNNOVATE trial,^[16]^ we opted for administrating ibrutinib and rituximab combination and our patient has achieved complete remission according to the Task Force criteria from the 8th International Workshop for WM.^[1]^

In conclusion, we report an infrequent presentation of the extremely rare BNS and we propose a novel sequential chemoimmunotherapy approach aiming both at rapid disease control and deep and durable remission with minimization of induced toxicity. In the absence of randomized clinical trials, multidisciplinary and inter-institutional collaboration is considered essential to improve diagnosis and treatment outcomes for patients with BNS.

## Author contributions

**Conceptualization:** Maria Gavriatopoulou, Meletios-Athanasios Dimopoulos.

**Investigation:** Maria Gavriatopoulou.

**Methodology:** Maria Gavriatopoulou.

**Writing – original draft:** Maria Gavriatopoulou.

**Writing – review & editing:** Maria Gavriatopoulou, Ioannis Ntanasis-Stathopoulos, Lia-Angela Moulopoulos, Alexandros Manaios, Despoina Fotiou, Evangelos Eleutherakis-Papaiakovou, Charis Bourgioti, Evangelos Terpos, Efstathios Kastritis, Meletios-Athanasios Dimopoulos.
